# MiR-34a is up-regulated in response to low dose, low energy X-ray induced DNA damage in breast cells

**DOI:** 10.1186/1748-717X-8-231

**Published:** 2013-10-05

**Authors:** Luiza Stankevicins, Ana Paula Almeida da Silva, Flavia Ventura dos Passos, Evelin dos Santos Ferreira, Maria Cecilia Menks Ribeiro, Mariano G David, Evandro J Pires, Samara Cristina Ferreira-Machado, Yegor Vassetzky, Carlos Eduardo de Almeida, Claudia Vitoria de Moura Gallo

**Affiliations:** 1 Departamento de Genética, Universidade do Estado do Rio de Janeiro, Instituto de Biologia Roberto Alcantara Gomes, 20550-013 Rio de Janeiro, Brazil; 2 Departamento de Biofísica, Laboratório de Ciências Radiológicas, Universidade do Estado do Rio de Janeiro, Instituto de Biologia Roberto Alcantara Gomes, 20550-013 Rio de Janeiro, Brazil; 3 Departamento de Biologia Celular, Embriologia e Genética, Universidade Federal de Santa Catarina, 88040-900 Florianopolis, Santa Catarina, Brasil; 4 Departamento de Biologia Geral, Universidade Federal Fluminense, Instituto de Biologia, 24020-141 Niterói, Rio de Janeiro, Brazil; 5 Université Paris-Sud 11 CNRS UMR 8126 «Signalisation, noyaux et innovations en cancérologie», Institut de Cancérologie Gustave-Roussy, Université Paris-Sud 11, F-94805 Villejuif cedex, Paris, France; 6 Departamento de Genética/UERJ, Rua São Francisco Xavier, 524/sala 525-6, Maracanã, 20 550-013 Rio de Janeiro, RJ, Brazil

**Keywords:** MicroRNA, Apoptosis, miR-34a, Breast cell lines, Breast cancer

## Abstract

**Background:**

MicroRNAs are non-coding RNAs involved in the regulation of gene expression including DNA damage responses. Low doses of low energy X-ray radiation, similar to those used in mammographic exams, has been described to be genotoxic. In the present work we investigated the expression of miR-34a; a well described p53-regulated miRNA implicated in cell responses to X-ray irradiation at low doses.

**Methods:**

Non-cancerous breast cell line MCF-10A and cancerous T-47D and MCF-7 cell lines were submitted to a low-energy X-ray irradiation (ranging from 28–30 Kv) using a dose of 5 Gy. The expression level of miR-34a, let-7a and miR-21 was assessed by qRT-PCR at 4 and 24 hours post-irradiation. DNA damage was then measured by comet assay and micronuclei estimation in MCF-10A and MCF-7 cell lines, where an increase of miR-34a levels could be observed after irradiation. The rate of apoptotic cells was estimated by nuclear staining and fluorescence microscopy. These experiments were also performed at low doses (3; 12 and 48 mGy) in MCF-10A and MCF-7 cell lines.

**Results:**

We have observed an increase in miR-34a expression 4 hours post-irradiation at 5 Gy in MCF-10A and MCF-7 cell lines while its level did not change in T-47D, a breast cancer cell line bearing non-functional p53. At low doses, miR-34a was up-regulated in non-tumoral MCF-10A to a higher extent as compared to MCF-7. MiR-34a levels decreased 24 hours post-irradiation. We have also observed DNA damage and apoptosis at low-energy X-ray irradiation at low doses and the high dose in MCF-10A and MCF-7 4 and 24 hours post-irradiation relative to the mock control.

**Conclusion:**

Low energy X-ray is able to promote DNA strand breaks and miR-34a might be involved in cell responses to low energy X-ray DNA damage. MiR-34a expression correlates with X-ray dose, time after irradiation and cell type. The present study reinforces the need of investigating consequences of low dose X-ray irradiation of breast cells.

## Background

MicroRNAs (miRNAs) constitute a class of evolutionarily conserved small non-coding RNAs which regulates gene expression at the post-transcriptional level. They were at first assigned to target complementary sequences in the 3′UTR of mRNAs, only requiring a continuous base-pairing of miRNA nucleotides 2 to 8, known as the seed sequence to subsequently direct mRNAs for translational inhibition and decay [1]. Later, recognition sites located outside 3′UTR have been observed by computational tools and validated by functional approaches [2,3], rendering these small RNAs to be involved in the regulation of virtually all cellular processes [4,5]. The global action of miRNAs is to repress gene expression and, by this way, they are involved in important cellular processes including DNA damage response (DDR) [6,7]. Ionizing radiation such as X-rays can harm cells by direct DNA breakage or indirectly through the creation of free radicals which will contribute to increase and prolong DNA damage [8]. Increasing evidence indicates that such injurious effects are not linear with the radiation dose mainly due to different cellular mechanisms to adapt or die, which are highly dependent on cell type and cell environmental conditions [8]. Hence, irradiated cells may activate DNA repair system or apoptosis. Initial cell responses to genotoxic stress occur through molecular sensors, usually kinases that trigger DDRs. For example, the ATM-mediated DDR activates the tumor suppressor protein p53, a transcription factor critical for genomic stability, regulating cell cycle progression and DNA repair, as well as apoptosis. In this same sense, it is expected that miRNAs are involved in mechanisms such those regulated by p53 [9,10]. Apoptosis and DNA repair are intricately connected with DNA damage and cancer. Irradiation leads to a massive change in miRNA expression pattern [11,12], unfortunately the roles of specific miRNAs in radiation response are not yet clearly identified. We decided to concentrate on three miRNAs, miR-34a, let-7a and miR-21 since they are consistently associated with the modulation of cell damage response pathways [13]. MiR-34a is a direct p53 target gene and its ectopic expression induces apoptosis, cell-cycle arrest in G1 or senescence [14]. *In vitro* experiments using *C elegans* and breast cancer cells as models showed that loss of function mutations in miR-34a gene generated an abnormal cellular survival response to radiation [15]. Validated miR-34a targets include several genes involved in DDR as Bcl-2, Notch1, Cyclin D1, Cyclin E2, CDK4, MET and SIRT1 [16-18], suggesting that miR-34a may serve as a marker of radiation injury and as a therapeutic target [14,19]. Let-7a is a member of a family which comprises 12 miRNAs with tumor suppressor activities that can be regulated in response to ionizing radiation. Among let-7a targets there are molecules involved in such important cellular activities as proliferation (K-ras; c-myc; E2F2) and cell cycle control (Cdc25a; Cyclin D1). Let-7a is usually down-regulated after ionizing radiation exposure, however its overexpression can increase radiosensitivity *in vivo* and in different tumor types mainly by downregulation of K-Ras [20,21]. Finally, miR-21, classified as an oncogenic miRNA, was described as a negative regulator of some suppressor genes related to proliferation, apoptosis and invasion such as PTEN, PDCD4, Tropomyosin-1 and Bcl-2 [22-24]. MiR-21 is often up-regulated in tumors and its overexpression is associated with a more proliferative and aggressive phenotype [25]. *In vivo* and *in vitro* studies suggest a role for miR-21 in tumor initiation and progression and as a possible diagnostic and prognostic marker for human malignancies. In breast cancer, miR-21 knockdown cells can trigger apoptotic cell death followed by a decrease in cell proliferation suggesting a function as anti-apoptotic factor [26]. MiR-21 is usually up-regulated after irradiation and its inactivation can contribute to radiation induced apoptosis [27,28]. Several miRNAs with aberrant expression are present ubiquitously in breast and other cancers. Microarray analysis shows a global change in miRNA expression in the presence of genotoxic agents including ionizing radiation [29]. To test the hypothesis that miR-34a is involved in the DDR after X-ray irradiation of breast cells, we determined relative expression of miR-34a, let-7a and miR-21, in the non-cancerous breast cell line MCF-10A and the breast cancer cell lines MCF-7 and T47-D, 4 and 24 hours after X-ray exposure at a high dose (5 Gy). We have also applied X-ray irradiation doses rate and energy equivalent to those utilized in mammographic exams, usually 10 mGy/s for 28 kV [30] in breast cells MCF-10A and MCF-7. Our results show an overexpression of miR-34a in the non-cancerous MCF-10A cells in response to DNA damage caused by low-doses of X-ray radiation.

## Materials and methods

### Cell culture

The human breast adenocarcinoma cell line MCF-7, the ductal carcinoma cell line T-47D and the non-cancerous epithelial breast cell line MCF-10A were obtained from David Cappellen and Nancy Hynes (Friedrich Miescher Institute for BioMedical Research, Novartis Research Foundation, Basel, Switzerland). The MCF-7 and T-47D cells were cultured in RPMI 1640 supplemented with 10% fetal bovine serum (FBS) and 1% penicillin, streptomycin. MCF-10A cells were maintained in DMEM/F12 supplemented with 10% FBS, hydrocortisone 0.5 μg/mL, insulin 10 μg/mL, EGF 20 ng/mL and 1% penicillin, streptomycin. The culture medium and FBS were purchased from Life Technologies (Carlsbad, CA, USA), all others supplements were from Sigma Aldrich (St Louis, MO, USA). Cultures were routinely checked for mycoplasma contamination.

### Irradiation of cells

Cells in the logarithmic growth phase were submitted to X-ray irradiation with a Philips X-ray tube (PW 2185/00 Side window) with Mo anode and 0.03 mm Mo filter, employed in mammography dosimeters calibration. After irradiation at 3; 12 and 48 mGy/28 kV and 5 Gy/30 kV, the cells were incubated for 4 and 24 hours in cultured medium, at 37°C, under 5% of CO_2_. A mock control performed with cells under the same conditions but the irradiation was added in all experiments.

### Comet assay (single cell electrophoresis assay)

Alkaline comet assay was performed according to the method described elsewhere [31] with modifications. Aliquots containing 2.5 x 10^5^ cells were pelleted and resuspended in 10 μL of PBS 1X (60 mM NaCl; 0.2 mM KCl; 0.1 mM Na_2_HPO_4_; 0.1 mM KH_2_PO_4_, pH 7.4). Briefly, 120 μL of a 0.5% low melting point agarose kept at 37°C were added to these cell aliquots and then immediately distributed on glass slides previously covered with a thin layer of 1.5% normal melting point agarose. After upper agarose solidification, the slides were transferred to a lysis solution (2.5 M NaCl; 10 mM Tris; 100 mM EDTA; 10% DMSO; 1% lauryl sarcosinate; 1% triton X-100; pH 10) at 4°C for 12 h and kept on electrophoresis alkaline buffer (0.3 M NaOH; 1 mM EDTA; pH 13) for 20 minutes before running electrophoresis at 25 V/300 mA for 20 minutes. After electrophoresis, DNA was silver stained according to [32,33]. A total of 100 cells were analyzed per slide and visually classified into four categories according to the degree of DNA damage, where 0 corresponds to no damage and 4 to a highly damaged DNA. The percentage of tail DNA was assigned by summing the total number of cells containing tail DNA, multiplied by its respective category number. All experiments were performed in triplicate and repeated at least two times.

### Apoptosis and micronuclei estimation

For apoptosis and micronuclei estimation, MCF-10A and MCF-7 cells were cultured as described before. Cells were submitted to a 3; 12 and 48 mGy/28 kV and 5 Gy/30 kV X-ray irradiation and incubated for 4 and 24 hours, at 37°C, under 5% of CO_2_. Then, cells were collected and the evaluation of nuclei morphology was performed by fluorescence microscopy, using HOECHST 33258 staining. The micronuclei assay was done based on Kirsch-Volders et al. [34]. The number of apoptotic and micronucleated cells was scored for each condition. A total of 100 nuclei were counted per slide. All experiments were performed in triplicate and repeated at least two times.

### RNA extraction and quantitative RT-PCR

Total RNA samples were obtained using Trizol reagent (Life Technologies; Carlsbad, USA), according to the manufacturer’s protocol. Reverse transcriptase reaction and quantitative PCR were performed using NCode VILO miRNA cDNA Synthesis Kit (Life Technologies; Carlsbad, USA). Real-time PCR was done using SYBR green reagents (Life Technologies; Carlsbad, USA). Primer sequences used in qPCR reaction were: β-actin(F) 5′-CATCGAGCACGGCATCGT-3′; β-actin(R) 5′-GCCTGGATAGCAACGTACAT-3′as loading control; miR-34a(F) 5′-GGCAGTGTCTTAGCTGGTTGT-3′; let-7a(F) 5′-CCGCTGAGGTAGTAGGTTGTATAGTT-3′; miR-21(F) 5′-GGCTAGCTTATCAGACTGATGTTGA-3′; with the universal reverse primer provided on miRNA cDNA synthesis kit. The reaction conditions were as following: denaturing at 95°C for 2 min and 40 cycles of denaturing at 95°C for 15 sec and annealing at 60°C for 60 sec followed by a melting curve. All experiments were done in triplicate and repeated at least two times. Relative expression was analyzed using the ΔΔCt method [35,36].

### Statistical analysis

All statistical analysis were performed on the irradiated samples of each type of cell line in comparison to its corresponding mock control using paired t-test with 95% confidence intervals.

## Results

**Figure 1:**
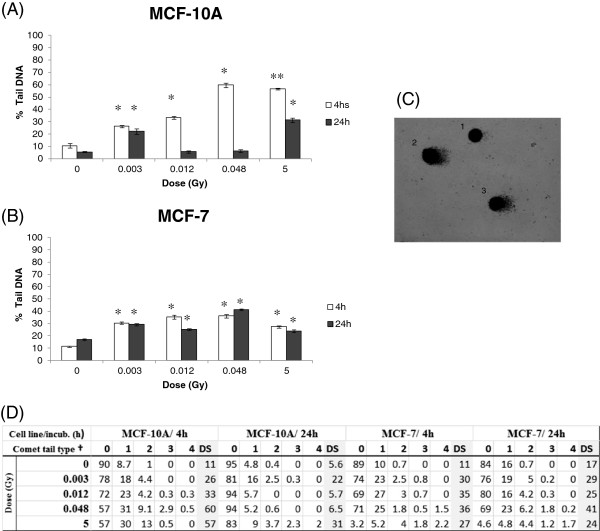
**Single cell electrophoresis assay performed after 4 and 24 hours incubation on X-ray irradiated cells at 0.003; 0.012; 0.048 Gy/28 kV and 5 Gy/30 kV.** The percentage of DNA strand breaks observed in the mammary epithelial cell lines **(A)** MCF-10A and **(B)** MCF-7 was analyzed according to the length and thickness of comet tail and classified into four categories from 1–4 representing increasing tail intensities. The percentage of tail DNA was assigned by summing the total number of cells containing tail DNA, multiplied by its respective category number. Total analyzed cells per slide 100. **(C)** Example of silver stained of a nuclei with no visible lesion (1) and a tail DNA classified as lesion type 2 (2, 3) * significant difference in comparison irradiated versus non-treated cells (Student’s t test * P ≤ 0.05; ** P ≤ 0.001). **(D)** Table showing the percentage of cells detected in each category of damage. † Comet tail type: 0 corresponds for no visible DNA breaks and the categories 1 to 4 represents increasing amounts of DNA breaks. DS stands for Damage Score and corresponds to the total percentage of tail DNA.

**Figure 2:**
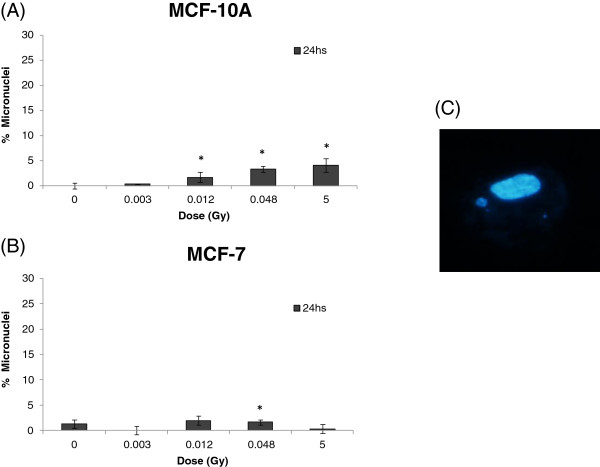
**Percentage of micronuclei formation, observed by fluorescent microscopy (1000x) after Hoechst staining. (A)** MCF-10A and **(B)** MCF-7 cell lines 24 hours after 0.003; 0.012; 0.048 Gy/28 kV and 5 Gy/30 Kv X-ray irradiation. **(C)** Example of a cell containing a micronucleus formed after irradiation. Paired t-test with 95% confidence intervals were performed on the irradiated samples of each type of cell line in comparison to its corresponding mock control. Student’s t test * P ≤ 0.05.

**Figure 3:**
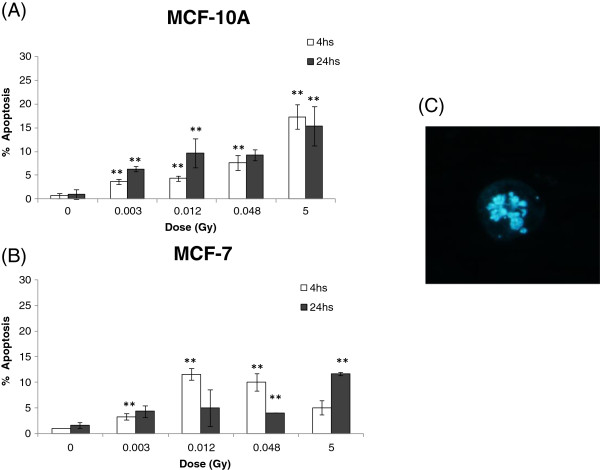
**Percentage of cells containing apoptotic features, observed by fluorescent microscopy (1000x) after Hoechst staining. (A)** MCF-10A and **(B)** MCF-7 cell lines 4 and 24 hours after 0.003; 0.012; 0.048 Gy/28 kV and 5 Gy/30 Kv X-ray irradiation **(C)** Example of an apoptotic cell displaying a perturbation in its nuclear envelope. Paired t-test with 95% confidence intervals were performed on the irradiated samples of each type of cell line in comparison to its corresponding mock control. (Student’s t test * P ≤ 0.05; ** P ≤ 0.001).

**Figure 4:**
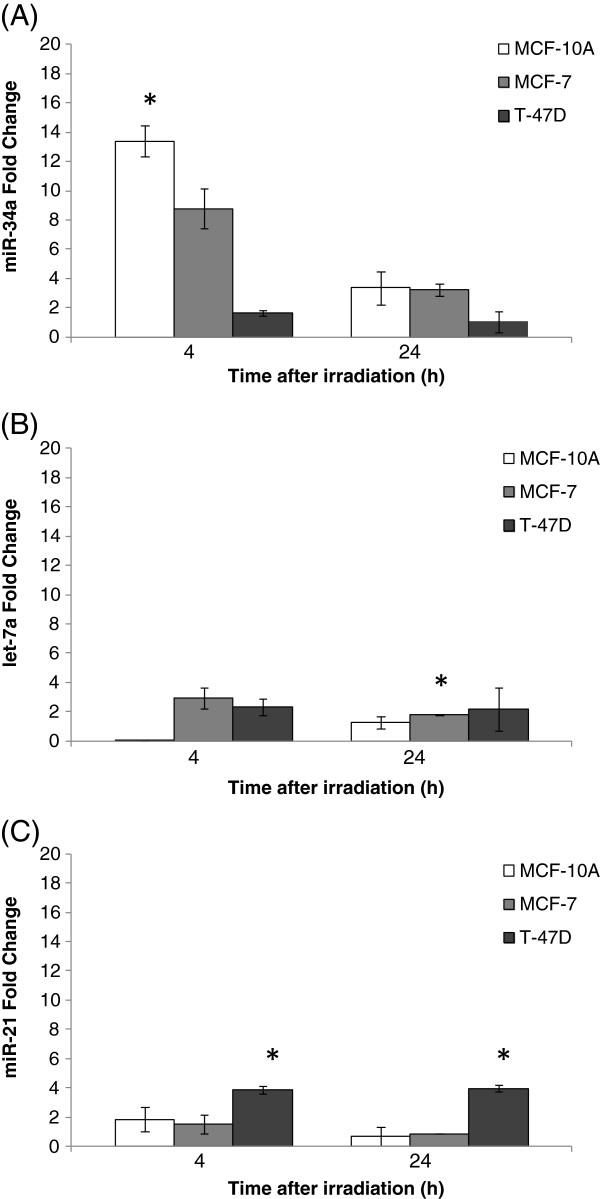
**microRNA expression by qRT-PCR in MCF-10A, MCF-7 and T-47D cell lines, assessed 4 and 24 hours after 5 Gy/30 Kv X-ray irradiation event.** Relative expression of miRNAs **(A)** miR-34a, **(B)** let-7a and **(C)** miR-21 was calculated by ΔΔCT method and the acquired fold change values are relative to their respective non-treated mock control. * significant difference in comparison irradiated versus non-treated cells (Student’s t test P ≤ 0.05).

**Figure 5:**
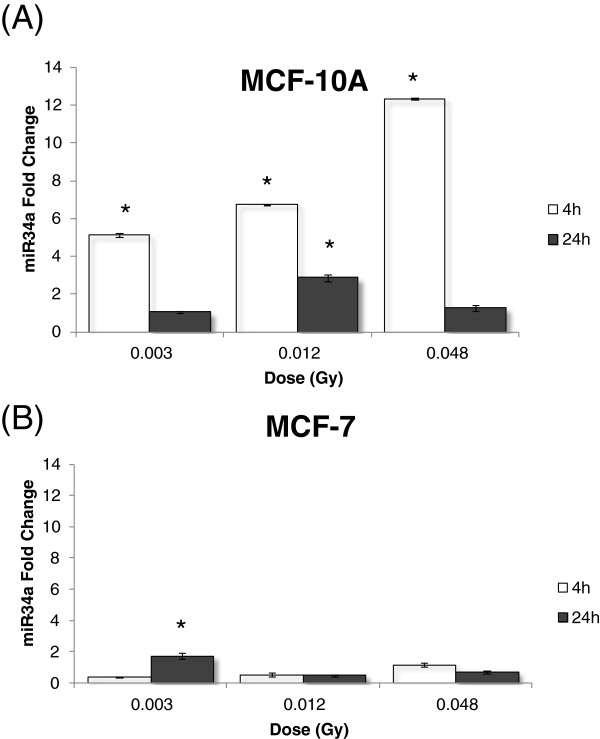
**miR-34a expression by qRT-PCR in (A) MCF-10A and (B) MCF-7cell lines, assessed 4 and 24 hours after X-ray irradiation at 0.003; 0.012 and 0.048 Gy/28 Kv conditions.** Relative expression of miR-34a was calculated by ΔΔCT method and the acquired fold change values are relative to their respective non-treated mock control. * significant difference in comparison irradiated versus non-treated cells (Student’s t test P ≤ 0.05).

To estimate DNA damage in different X-ray doses we first performed the comet assay with irradiated non-cancerous breast cells MCF-10A and cancerous MCF-7 cells, using low-energy (28/30 kV) X-ray irradiation at low doses (3; 12 and 48 mGy) and at a high dose (5 Gy). As shown in Figure 1, DNA lesions were observed at all applied doses 4 hours after irradiation. MCF-10A presented a higher level of DNA lesions, approximately 60% of damaged cells at 5 Gy compared to 30% in MCF-7 cells (Figure 1A and B). At 24 hours, a persistence of DNA strand breaks was observed in MCF-7 but not in MCF-10A (Figure 1A and B). Both cell lines showed low levels of micronucleated cells at all analyzed irradiation conditions and time points, with MCF-10A cells showing almost no detectable micronuclei. MCF-7 cells presented approximately 5% of micronucleated cells at 24 hours after irradiation with 5 Gy, the highest dose (Figure 2A and B). Nevertheless, we could detect the occurrence of apoptotic cells in both cell lines at 4 and 24 hours after irradiation, although neither dose- nor time-dependent (Figure 3A and B). We have next performed qRT-PCR in order to detect alterations in miR-34a, let-7a and miR-21 known to be involved in DDR [13]. We have first used a high dose (5 Gy) to irradiate cells and we observed that miR-34a was the most up-regulated one, with approximately increase of 13 times fold change in MCF-10A and 9 times fold-change in MCF-7, 4 hours after 5 Gy X-ray irradiation (Figure 4 A-C). At 24 hours, miR-34a levels lowered, relative to the expression observed at 4 hours, showing an approximately 4 times fold-change when compared to the non-treated control, in both cell lines. We also investigated the miRNAs expression levels in T-47D cell line and the miR-34a expression was not altered (Figure 4A). We have next tested whether miR-34a is also up-regulated in breast cells in response to low doses of X-ray irradiation. We have applied doses and energy equivalent to that used in mammographic exams, approximately 10 mGy/28 kV [30]. Therefore, we have irradiated exponentially growing cells with doses 3; 12 and 48 mGy and quantified miR-34a expression at time points 4 and 24 hours. As demonstrated in Figure 5A, miR-34a up-regulation is clearly observed in MCF-10A cells at 4 hours after irradiation in a dose-dependent fashion. Interestingly, with MCF-7 this same effect is not observed (Figure 5B). Since an important miR-34a up-regulation of 8 times fold-change was detected in MCF-7 cells at a dose of 5 Gy (Figure 4A), our results suggest that in this cancer cell line higher doses are necessary to induce miR34a expression.

## Discussion

Recent findings regarding the use of low-energy X-ray radiation at low doses commonly employed in mammographic exams, indicate that such irradiation is potentially more harmful to cells than considered by physicians. In 2004, Heyes and Mill [30], by relative biological effectiveness studies (RBE), demonstrated that the low-energy X-rays used in mammography (approximately 29 kV) increased four-fold the risk of mutational DNA damage than higher energy γ-radiation. The authors concluded that the risks of radiation-induced breast cancers for low energy X-rays are underestimated by the same factor. The biological effects caused by ionizing radiation are explained by two phenomena: one of a direct target hit, where the nuclear DNA is the major target molecule and the other by non-targeted effects or indirect actions of radiation. Low doses at low-energy radiation are characterized by a high indirect activity through reactive oxygen species generation and bystander effects. These effects persist for long periods, whereas DNA breaks induced by direct radiation are repaired relatively quickly [8,30,37]. Therefore, both direct and indirect ionizing radiation effects result in DNA damage and in DNA damage cell responses (DDRs) [38]. MicroRNAs have been described as a class of regulatory molecules which can modulate cell physiology and are associated to several pathways including G1 arrest, DNA repair and apoptosis. They have been also described to be induced by X-rays evoking a role of intermediate molecules in the regulation of DDRs [39,40]. Due to the widespread utilization of mammographic exams, the possible relationship between miRNAs expression and DNA damage caused by low energy, low-dose X-ray irradiation is an important matter of study. We have designed experiments to first demonstrate the presence of DNA lesions during low energy, low-dose X-ray irradiation of mammary cells in culture and then proceed to the determination of miRNAs expression in these conditions. Comet assay and micronuclei estimation were performed after irradiation with the cells MCF-10A and MCF-7, a non-cancerous and a cancerous cell line, respectively. The comet assay permits to detect DNA single-strand breaks, alkali-labile sites and double strand breaks associated with incomplete excision repair sites [31] while the micronuclei detects the occurrence of persistent double strand breaks [41]. Although not linearly related to the applied doses, at 4 hours after X-ray irradiation with 3; 12 and 48 mGy/28 kV and 5 Gy/30 kV doses, an increase in DNA lesions was clearly observed in the comet assay. Moreover, the cell line MCF-10A presented higher levels of DNA breaks than MCF-7 cells. At 24 hours, however, MCF-10A cells apparently recovered DNA integrity, at least at low doses, while MCF-7 presented persistent DNA lesions. This is in agreement with the data of Francisco et al., 2008 [42] who demonstrated that MCF-7 cells tend to accumulate more DNA lesions than MCF-10A after γ-radiation exposure. We then analyzed the expression levels of three miRNAs: miR-34a, let-7a and miR-21. The miR-21 is considered an oncogenic miRNA and is frequently over-expressed in several types of cancer; conversely, let-7a and miR-34a function as tumor suppressors and are frequently down-regulated in cancer. MiR-34 family expression is induced by p53 which is a key protein regulating different forms of cellular stress, including X-ray irradiation [43]. Among the analyzed miRNAs, miR-34a showed to be the most over-expressed after 5Gy irradiation in both MCF-10A and MCF-7 cells. MiR-34a expression was also measured after the low-dose irradiation in MCF-10A and MCF-7 cell lines. Interestingly miR-34a expression appears to correlate with the applied dose and the apoptosis level in MCF-10A. Hence, this miRNA might act as an irradiation biomarker in normal cells. MCF-7 cells presented a significant miR-34a up-regulation at the lower dose 3 mGy. Some cell lines have been observed to exhibit hypersensitivity to low radiation doses [44]. Although the direct effect of radiation in the hit DNA molecule is proportional to the dose, effects occurring in non-targeted molecules cannot be predicted in a dose–response fashion. Usually, the enhanced sensitivity at low doses of ionizing radiation reflects the failure of the repair machinery to fully arrest the progression of the cell cycle, preventing unrepaired DNA breaks, from undergoing cell division [44]. This may be the case with the MCF-7 cells.

Women at high risk of breast and ovarian cancers are usually more susceptible to radiation-induced cancer because most of tumor suppressor genes implicated in breast cancer susceptibility are also implicated in the radio-induced DNA damage repair and signaling such as *BRCA1/2*, *TP53* and *ATM*[45,46]. The results of a European cohort study associates diagnostic radiation before the age of 30 with an increased risk of breast cancer among BRCA1/2 mutation carriers [47]. Unfortunately, this is the group of women most exposed to low energy radiation, since they are subject to annual prophylactic screening by the age of 30–35, while in the rest of population, mammography is recommended at age 40 [48]. Our observations may help to understand the effects of low energy X-ray irradiation in healthy women and breast cancer patients.

## Conclusion

Our results indicate that miR-34a is up-regulated after X-ray irradiation in P53 positive normal and cancer breast cell lines. MiR-34a might be involved in breast cell responses to low dose X-ray DNA damage. The present study reinforces the need of investigating consequences of low dose X-ray irradiation of breast cells.

## Competing interests

The authors declare that there are no conflicts of interest.

## Authors’ contributions

LS carried out the molecular analysis, cell cultures, irradiation planning, result analysis and drafted the manuscript. APAS carried out cell cultures and morphological assays. FVP participated in the morphological assays. ESF participated in the miRNA expression assays. MCMR carried out technical assistance in the morphological determinations. MGD and ELP were responsible for cell irradiation, irradiator calibration and dose calculations. SCFM participated in technical assistance in the morphological assays and helped to draft the manuscript. YV participated in the study design, critical revision of manuscript. CEA participated in the study design, support concerning technical questions and critical revision of the manuscript. CVMG conceived the study, participated in its design and coordination, and wrote the manuscript. All authors read and approved the final manuscript.
